# High-Temperature Tensile and Creep Behavior in a CrMoV Steel and Weld Metal

**DOI:** 10.3390/ma15010109

**Published:** 2021-12-24

**Authors:** Yan Song, Mengyu Chai, Zelin Han, Pan Liu

**Affiliations:** School of Chemical Engineering and Technology, Xi’an Jiaotong University, Xi’an 710049, China; songyan1211@mail.xjtu.edu.cn (Y.S.); hanzelin@stu.xjtu.edu.cn (Z.H.); lp2441867383@stu.xjtu.edu.cn (P.L.)

**Keywords:** CrMoV steel, weld, tensile, creep, creep cavity

## Abstract

The 2.25Cr1Mo0.25V steel is a vanadium-modified 2.25Cr1Mo steel and is being widely used in the manufacture of heavy-wall hydrogenation reactors in petrochemical plants. However, the harsh service environment requires a thorough understanding of high-temperature tensile and creep behaviors of 2.25Cr1Mo0.25V steel and its weld for ensuring the safety and reliability of hydrogenation reactors. In this work, the high-temperature tensile and creep behaviors of base metal (BM) and weld metal (WM) in a 2.25Cr1Mo0.25V steel weldment used for a hydrogenation reactor were studied experimentally, paying special attention to its service temperature range of 350–500 °C. The uniaxial tensile tests under different temperatures show that the WM has higher strength and lower ductility than those of BM, due to the finer grain size in the WM. At the same time, the short-term creep tests at 550 °C reveal that the WM has a higher creep resistance than that of BM. Moreover, the creep damage mechanisms were clarified by observing the fracture surface and microstructures of crept specimens with the aid of scanning electron microscopy (SEM). The results showed that the creep damage mechanisms of both BM and WM are the initiation and growth of creep cavities at the second phase particles. Results from this work indicate that the mismatch in the high-temperature tensile strength, ductility, and creep deformation rate in 2.25Cr1Mo0.25V steel weldment needs to be considered for the design and integrity assessment of hydrogenation reactors.

## 1. Introduction

Hydrogenation reactors are the key equipment in petrochemical hydroreforming, hydrorefining, and other petrochemical plants. The safety of hydrogenation reactors is being given much attention because of the harsher service environment in the context of carbon dioxide emissions reduction. The reactors are generally exposed to high temperatures for a long time. For instance, the hydroreforming process requires a design temperature of the reactor of up to 500 °C and design pressure up to 5 MPa, which makes the high-temperature strength and creep properties of the reactor steels of primary interest.

Conventional high-strength low alloy Cr–Mo steels, such as 2.25Cr1Mo steel, have been widely used in the manufacture of pressure vessels in the petrochemical industry for several decades. Nowadays, an upgraded version of 2.25Cr1Mo steel, i.e., 2.25Cr1Mo0.25V steel, has been developed and is being employed in the fabrication of new heavy-wall hydrogenation reactors [1]. This is because the addition of vanadium leads to higher strength and enhanced resistance to hydrogen attack and high temperature damage as compared to traditional 2.25Cr1Mo steel [1,2]. The use of the improved material allows the lightweight design of the hydrogenation reactor, which can significantly reduce the manufacturing cost [3]. On the other hand, welding is widely used for manufacturing pressure vessels. The weld is generally regarded as the weak link of the components operated at high temperatures because of the mismatch of microstructures and, therefore, the mechanical properties. Thus, to ensure the safety and reliability of hydrogenation reactors, the high-temperature strength and creep performance of the 2.25Cr1Mo0.25V steel and weld metal must be understood.

So far, much work has been performed to investigate the microstructures and tensile properties [3,4,5,6], reheat cracking behavior [7,8], fatigue behavior [9,10], and hydrogen-induced damage at high temperature [11] of 2.25Cr1Mo0.25V steel. For instance, Fu et al. [4] and Jiang et al. [5] investigated the influences of different heat treatment processes on the microstructure and mechanical responses of 2.25Cr1Mo0.25V steel. Results from their work showed that the time of tempering plays a vital role in regulating the microstructure and tensile strength of the material. It was also reported that the hot bending process during the manufacture of pressure vessels resulted in the degradation of mechanical properties of 2.25Cr1Mo0.25V steel due to significant plastic deformation caused by plate bending [6,11]. Furthermore, high-temperature hydrogen damage can cause significant reduction of ductility in 2.25Cr1Mo0.25V steel [11]. In a work concerning the fatigue behavior, Peral et al. [9] studied the effect of pre-charging hydrogen on the fatigue crack growth rate of quenched and tempered 2.25Cr1Mo and 2.25Cr1MoV steels. Their results demonstrated that the hydrogen effect on the crack propagation rate of 2.25Cr1MoV steel was notably lower than that of 2.25Cr1Mo steel because of the uniform dispersion of vanadium carbides, which act as strong traps for hydrogen. Studies concerning the microstructures and mechanical properties of 2.25Cr1Mo0.25V steel welded joints have also been performed. For instance, Yang et al. investigated the tensile behaviors of different zones in a CrMoV steel weldment by designing and using miniature tensile specimens at room temperature [12]. The results showed that there was a significant difference between the base metal and weld metal of the weldment, including both the microstructure and tensile strength. A more recent work performed by Li et al. [13] also investigated the tensile and impact responses of 2.25Cr1Mo0.25V steel parent metal and weld at room temperature. Their results showed that the weld metal exhibited higher strength but lower toughness than the parent metal, which may be due to the presence of more carbides and inclusions in weld. Our recent work investigated the fatigue crack growth behaviors of different zones (i.e., parent metal, heat affected zone, and weld metal) in an as-received 2.25Cr1Mo0.25V steel weldment [10]. The results showed that the heat-affected zone exhibited a higher fatigue crack growth rate due to non-uniform microstructure with coarse grains, whereas the parent metal showed superior fatigue resistance. However, it is worth noting that few studies focus on the tensile properties of 2.25Cr1Mo0.25V steel and weld at high operating temperatures. Therefore, the high-temperature tensile behavior as well as the damage mechanism of 2.25Cr1Mo0.25V steel and its weld require an in-depth understanding for the design and reliability analysis of hydrogenation reactors.

On the other hand, the creep property has been a major concern in the design and integrity assessment of hydrogenation reactors. The creep phenomenon is a process where permanent deformation occurs and increases with time under a constant applied stress which is lower than the yield strength. Plenty of studies on creep performance of traditional 2.25Cr1Mo steel and its welded joint have been carried out so far, including creep deformation and rupture behaviors, damage mechanism, and life prediction models [14,15,16,17,18]. Unfortunately, only a few studies reported the creep behavior of different zones in 2.25Cr1Mo0.25V steel weldments [19,20,21]. It was found that the microstructure heterogeneity in the weld is likely to cause a complicated creep response compared to that of base metal [20]. Moreover, the initiation and growth of creep cavities and cracks dominated the creep damage in the welded joint, leading to the final rupture [19,21]. The limited work on creep study of 2.25Cr1Mo0.25V steel welded joints hinders a thorough understanding of the creep deformation behavior as well as the damage mechanism of the parent material and weld.

In the present study, the purpose is to experimentally investigate the high-temperature tensile and creep behaviors of base metal (BM) and weld metal (WM) in a 2.25Cr1Mo0.25V steel weldment. Multi-pass welding was performed on a steel plate with a thickness of 112 mm which is used for manufacturing the heavy-wall hydrogenation reactor. The round bar-shaped tensile and creep specimens were machined from the BM and WM of the weldment. High-temperature tensile tests were performed in air at the temperature range of 350–550 °C, paying particular attention to the operating temperatures of the hydrogenation reactor. Moreover, the creep deformation behavior of the BM and WM were examined at 550 °C. The difference in the tensile and creep deformation behaviors between BM and WM was discussed. Finally, the damage mechanism associated with cavity nucleation during creep was clarified. Results from this study can provide reference for the design and manufacture of heavy-wall hydrogenation reactors.

## 2. Materials and Methods

The experimental study was carried out on a 2.25Cr1Mo0.25V steel weldment, which was manufactured by Lanzhou LS Heavy Equipment Co., Ltd. (Lanzhou, China). The 2.25Cr1Mo0.25V steel plates with a thickness of 112 mm were welded together by the combined shielded metal arc welding (SMAW) and submerged automatic arc welding (SAAW) techniques. SMAW was first applied for root welding by using the direct current electrode positive (DCEP) polarity, and SAAW was used to perform the remaining passes. During the SAAW process, the welding current and arc voltage were maintained at approximately 580 A and 32 V, respectively. After welding, an appropriate post-weld heat treatment (PWHT) was carried out to relieve the residual stress and enhance the toughness of the weldment. Specifically, the weldment was first heated to 400 °C at the rate of 12.7 °C/min, then heated to 705 °C at the rate of 1.27 °C/min and maintained for 8 h, and finally cooled to room temperature at the rate of 0.9 °C/min. Finally, the weldment was analyzed using non-destructive X-radiography for detecting any welding defects, and no cracks or pores were found. The main chemical compositions of the 2.25Cr1Mo0.25V steel BM and WM are given in Table 1.

Metallographic specimens of 10 × 10 × 10 mm were extracted to investigate the microstructures of the BM and WM. The metallographic specimen surfaces were gradually wet polished with grinding papers up to 1500 grit and then mirror-polished using a 1 μm diamond paste. The etching of the specimen surface was performed by using a 5% Nital solution according to the ASTM E407-07 standard [22]. Microstructures of the BM and WM were observed by using a field emission scanning electron microscope (FESEM, MAIA3LMH, TESCAN, Brno, Czech Republic). Moreover, to study the high-temperature tensile and creep damage mechanisms, the fracture surface morphologies of fractured specimens and the distribution of cavities of crept specimens were observed by using the FESEM.

**Figure 1:**
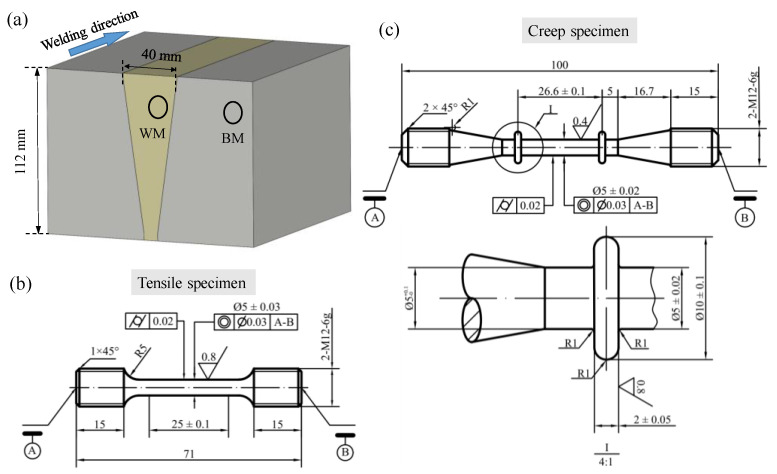
(**a**) Schematic for extracting specimens from the 2.25Cr1Mo0.25V steel weldment. Dimensions of (**b**) tensile specimen and (**c**) creep specimen. All units are in mm.

Cylindrical uniaxial tensile and creep test specimens, including BM and WM specimens, were machined from the 2.25Cr1Mo0.25V steel weldment in the welding direction, as shown in Figure 1. Specifically, the gauge section was 25 mm in length and 5 mm in diameter, for tensile specimens, and 24.6 mm in length and 5 mm in diameter, for creep specimens. The loading direction was parallel to the welding direction for both tensile and creep tests.

The tensile tests were carried out in the temperature range of 350–550 °C using a hydraulic material testing machine (MTS, Eden Prairie, MN, USA) in accordance with the ASTM E8/E8M standard [23]. The selected temperature range is consistent with the design temperatures of hydrogenation reactors in hydroreforming, hydrorefining, and hydrocracking plants. Before tensile loading, the specimens were first heated to the test temperature and kept constant for 20 min in the unstrained state. During loading, the specimens were first strain-controlled up to approximately 1% at 2.5 × 10^−4^/s in order to determine the yield properties. Subsequently, the control mode was shifted to the displacement control with a rate of 4.5 mm/min (corresponding to the strain rate of 2.5 × 10^−3^/s) to determine the tensile strength until the final fracture occurred. The high-temperature tensile tests for both BM and WM specimens were repeated three times to ensure data consistency.

The short-term creep tests were carried out at 550 °C under constant load in air by using a creep testing machine equipped with a resistance heating furnace according to the ASTM E139-11 standard [24]. The creep tests were performed at 350 MPa, which was lower than the yield stress. The choice of the applied stress value was to balance the creep damage influence and the time cost. Prior to creep loading, the specimens were held at 550 °C for 1 h to ensure uniform temperature inside the specimens. For the duration of the creep tests, the temperatures were maintained within ±2 °C, and the creep strain was measured until rupture.

## 3. Results & Discussion

### 3.1. Microstructure Characteristics

**Figure 2:**
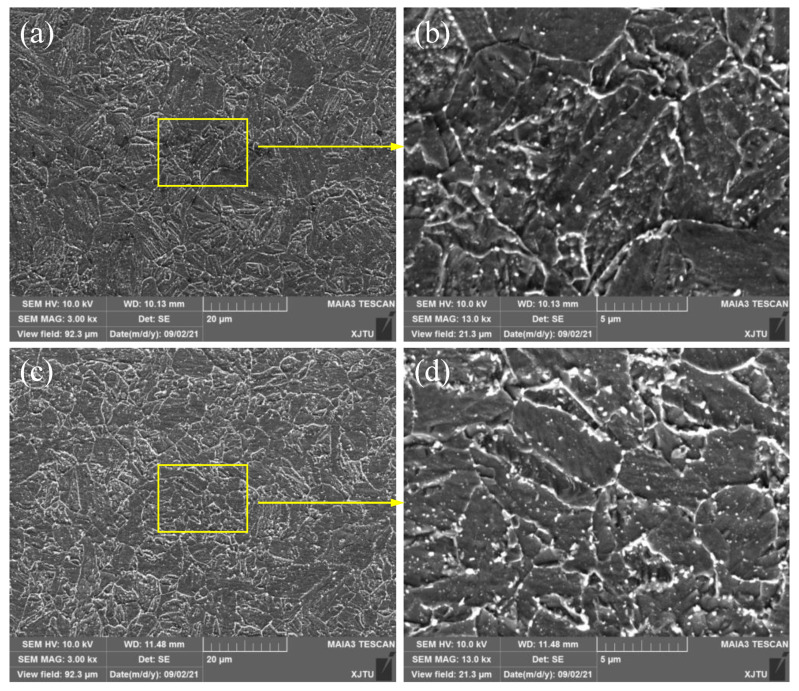
Microstructures of 2.25Cr1Mo0.25V steel (**a**,**b**) BM and (**c**,**d**) WM. The magnified SEM images (**c**,**d**) show the presence of a number of fine carbides in the grain boundaries and ferrite matrix.

The microstructural morphologies of the 2.25Cr1Mo0.25V steel welded joint after the PWHT process were observed and illustrated in Figure 2. Figure 2a shows the microstructure of the BM, which is mainly composed of bainite. As can be seen in Figure 2c, the microstructure of the WM is also bainite. However, it should be noted that the grain size of the WM is smaller than that of the BM, which is due to the grain refinement caused by the PWHT process. Moreover, higher magnification SEM images of the BM and WM in Figure 2b,d (Magnification 13,000×) show that a number of fine carbides disperse non-homogeneously in the grain boundaries and ferrite matrix.

### 3.2. Tensile Behaviors

#### 3.2.1. High-Temperature Tensile Properties

**Figure 3:**
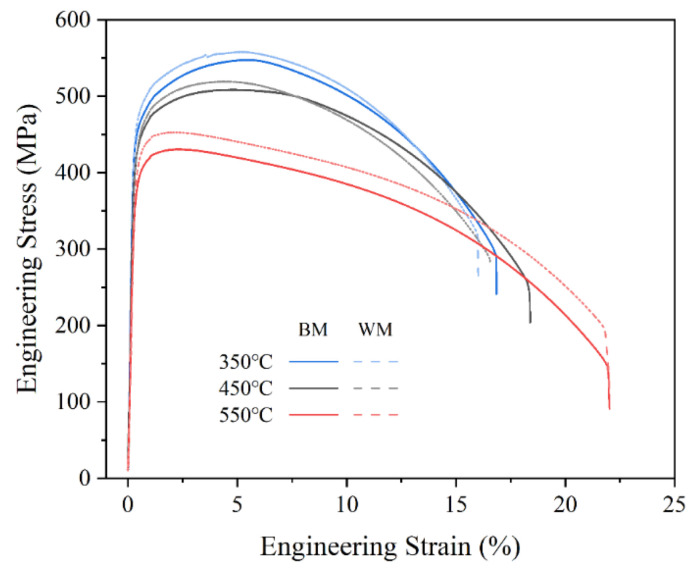
Engineering stress–strain curves of 2.25Cr1Mo0.25V steel BM and WM at elevated temperatures.

Figure 3 displays typical engineering stress–strain curves plotted from the load–displacement data acquired by tensile tests of the 2.25Cr1Mo0.25V steel BM and WM specimens at 350, 450, and 550 °C. In all cases, an obvious two-stage feature is observed, involving the elastic deformation stage and plastic deformation stage for all specimens, which indicates that the fracture modes of the BM and WM are ductile in nature. However, their tensile properties are different. Specifically, the WM exhibits a slightly higher tensile strength than the BM at all testing temperatures. Additionally, it can also be seen that there is a continuous softening behavior with an increase of testing temperature for both BM and WM specimens.

**Figure 4:**
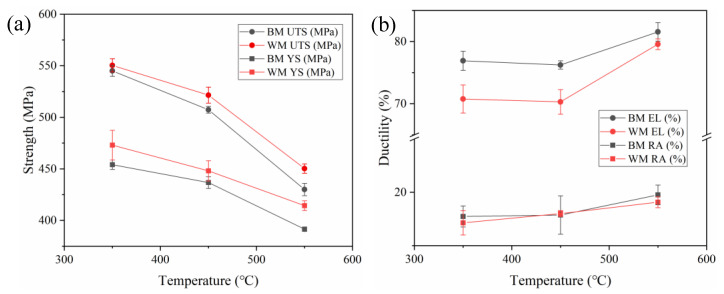
Variation of (**a**) strengths (YS and UTS) and (**b**) ductility (EL and RA) of 2.25Cr1Mo0.25V steel BM and WM as a function of temperature.

The yield strength (YS), which was measured at 0.2% offset of plastic strain, and the ultimate tensile strength (UTS) were determined from the engineering stress–strain curves. Additionally, the elongation (EL) and reduction of area (RA) were determined from the fractured tensile specimens in order to characterize the ductility. These tensile properties are shown in Table 2 and Figure 4. Substantial differences can be found between the values of strengths at various temperatures. For both BM and WM, the YS and UTS decrease monotonically with an increase of testing temperature, showing a general tendency of metallic materials. Specifically, the increase of temperature from 350 to 550 °C led to a 13.7% reduction in average YS and 21.2% reduction in average UTS for the BM, whereas these values were obtained as 12.4% and 18.2% for the WM, indicating that the strengths of the weld are relatively insensitive to the change in temperature. On the other hand, the WM obviously shows a higher YS and UTS than the BM. This observation can be attributed to the finer grain size in the WM. However, such a difference is insignificant. The difference between the YS values of the BM and WM at each temperature is less than 20 MPa. Moreover, the BM shows slightly higher values of EL and RA than the WM. This is reasonable, because the grain refinement can obviously enhance the strength of materials, whereas the ductility inevitably decreases [25]. In addition, despite the similar values of EL at 350 and 450 °C, the ductilities of both materials show an overall improvement with an increase in temperature, indicating a softening behavior with increasing temperature.

#### 3.2.2. Tensile Fracture Mechanisms

**Figure 5:**
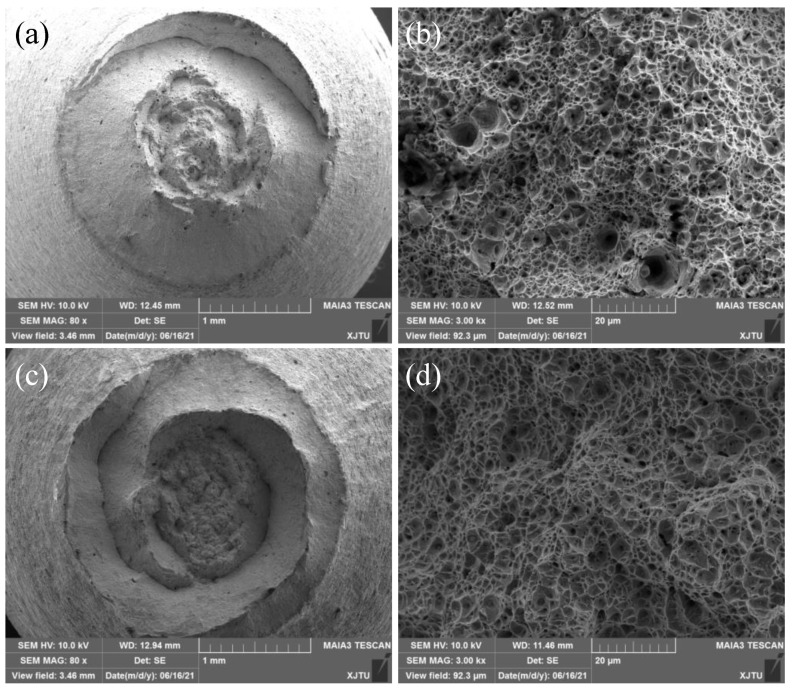
SEM images showing tensile fracture surfaces of (**a**,**b**) BM and (**c**,**d**) WM specimens at 350 °C.

**Figure 6:**
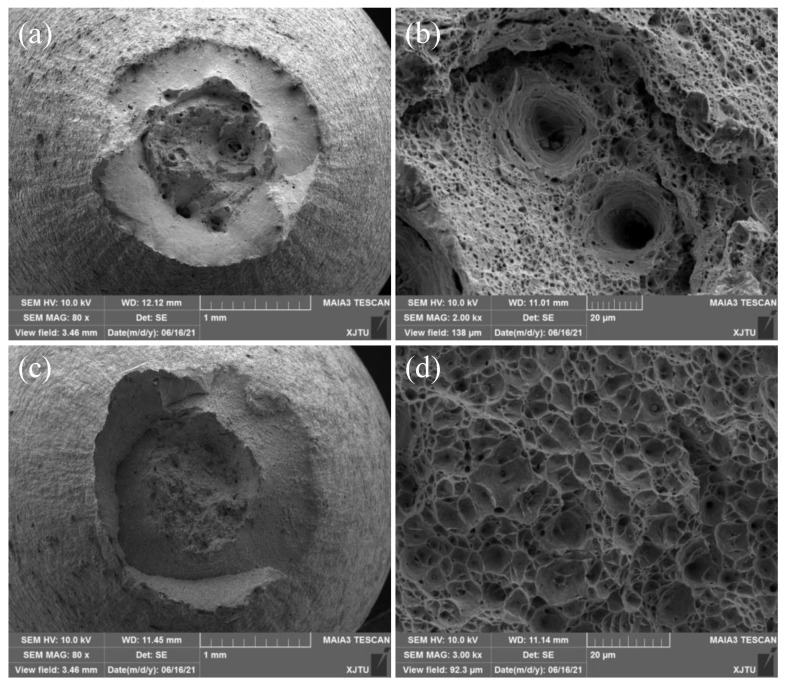
SEM images showing tensile fracture surfaces of (**a**,**b**) BM and (**c**,**d**) WM specimens at 550 °C.

To investigate the tensile fracture mechanisms of 2.25Cr1Mo0.25V steel BM and WM, the fracture morphology of fractured specimens tested under different temperatures was analyzed by SEM. Figure 5 and Figure 6 illustrate the fracture morphologies of BM and WM specimens tested at 350 and 550 °C, respectively. It is obvious that all of the BM and WM specimens exhibit transgranular ductile fracture with void nucleation, growth, and coalescence at different temperatures. In particular, the fracture surface of the BM consists of several primary dimples with large size and depth and a number of dimples with small size, as shown in Figure 5b and Figure 6b, whereas the fracture surface of the WM mainly consists of numerous small and shallow dimples, as presented in Figure 5d and Figure 6d. These results indicate that the BM is more ductile as compared with the WM, because more plastic strain and energy would be consumed during the formation of dimples with larger size. By comparing the overall fracture surfaces at 350 and 550 °C, a remarkable, larger reduction of area can be found on the fracture surfaces of specimens tested at 550 °C. Moreover, the size of dimples is larger in the fracture surface under 550 °C than that under 350 °C, suggesting an increase in ductility with increasing temperature.

### 3.3. Short-Term Creep Behaviors

#### 3.3.1. Creep Deformation Behavior

**Figure 7:**
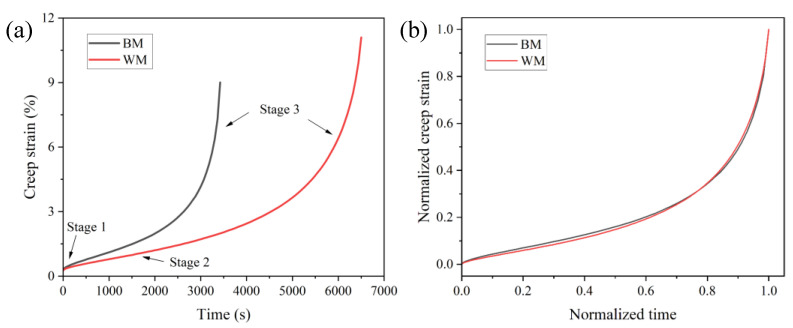
(**a**) Creep deformation curves of 2.25Cr1Mo0.25V steel BM and WM at 550 °C. (**b**) Normalized creep curves using the corresponding lifetime and creep strain.

Short-term creep tests were performed for 2.25Cr1Mo0.25V steel BM and WM specimens at 550 °C under 350 MPa. The complete variations of creep strain with respect to test time are depicted in Figure 7a. It is noted that the total creep strain increases with time. Moreover, the creep strain curves of both BM and WM specimens display three distinct damage stages according to the creep strain rate, i.e., the primary creep stage, the secondary/steady state creep stage, and the tertiary creep stage. The first stage occupies less than 10% of the whole creep life, and the corresponding creep rate reduces rapidly with time from the initial high values. Specifically, the primary creep strain can be estimated as the intercept between the steady state creep curve and the ordinate. In the secondary stage, the creep strain rate reaches its minimum value and keeps a steady state for a long duration. During this stage, a dynamic balance condition between the dynamic recovery through dislocation, annihilation, and rearrangement and work hardening is reached [26]. It can be noted from Figure 7a that the steady-state creep strain rate of the WM is apparently lower than that of the BM specimen because of the lower slope of creep curve in the secondary stage, indicating a higher creep deformation rate in the BM. In the tertiary stage, however, the dynamic balance state is disrupted with a rapid increase in creep strain rate until the specimen is fractured.

The comparison between the creep curve of the BM and WM shows that the creep lifetime of the WM specimen is significantly greater than that of BM, indicating a good creep damage resistance of WM in the 2.25Cr1Mo0.25V steel welded joint compared to BM. To further compare the creep deformation behaviors of BM and WM specimens, the creep strain curves were normalized using the corresponding lifetime and creep rupture strain, as presented in Figure 7b. It is interesting to note that the normalized creep curves of both BM and WM specimens are almost collapsed into one single curve. The creep curves of the BM and WM show a pronounced secondary and tertiary creep stage. Specifically, the secondary stage dominated the creep lifetime, being approximately 60% of the whole life. Moreover, the almost identical normalized creep curves suggest a similar creep damage mechanism contributing to creep failure of the BM and WM specimens.

#### 3.3.2. Creep Damage Mechanism

**Figure 8:**
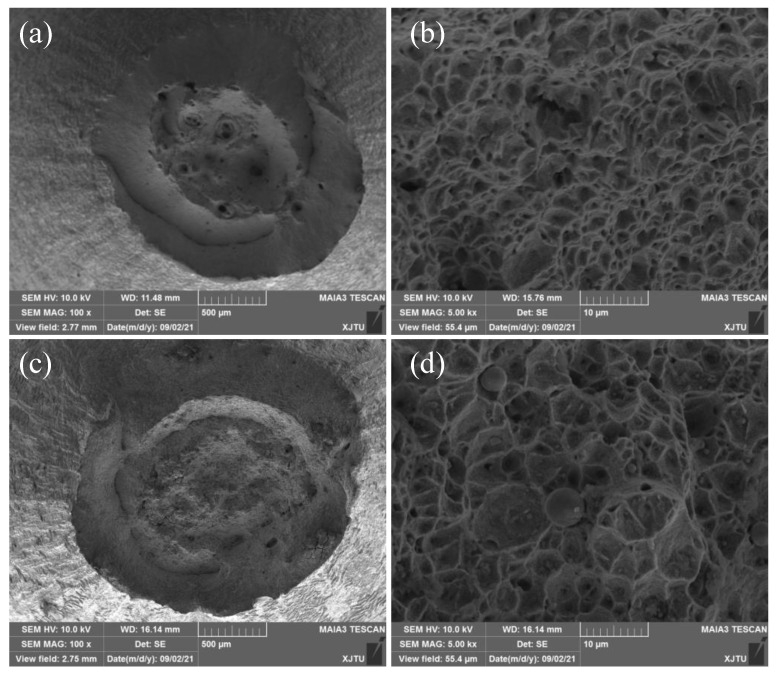
SEM images showing creep rupture surfaces of (**a**,**b**) BM and (**c**,**d**) WM specimens at 550 °C.

The creep fracture mode was investigated by observing the fracture morphology of 2.25Cr1Mo0.25V steel BM and WM specimens with the aid of SEM. Figure 8 illustrates the SEM fractographs of creep-ruptured BM and WM specimens under the stress of 350 MPa. It is apparent that both BM and WM specimens exhibit transgranular ductile fracture. Larger reduction of area can be found on the fracture surfaces of BM specimens, as shown in the overall fracture surfaces in Figure 8a,c. Moreover, several dimples with much larger size and depth can be seen in the macroscopic fracture surface of the BM (see Figure 8a) compared with the WM specimen (see Figure 8b). These results indicate that the BM is more ductile than the WM, which is in good agreement with the higher ductility of the WM obtained from high-temperature tensile tests (see Figure 4b). A large number of small dimples with equal-axis shape can be found in Figure 8b,d for both BM and WM specimens, suggesting that the normal stress provided by the applied stress plays a predominant role during the creep deformation and rupture process. Additionally, some inclusions can be found in the center of dimples on the fracture surface of WM, as displayed in Figure 8d.

**Figure 9:**
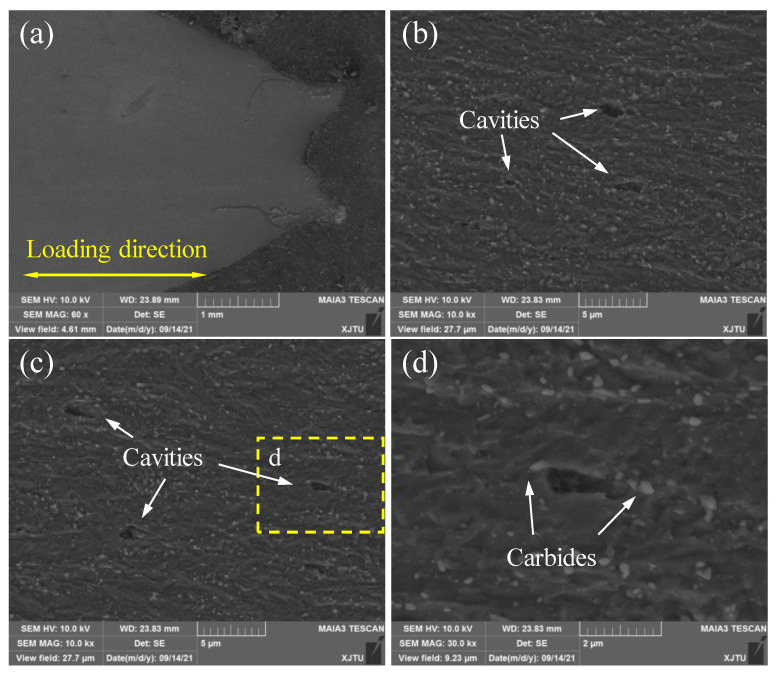
SEM images of the main damage zone of creep-ruptured BM specimen at 550 °C. (**a**) The overall micrograph of the longitudinal cross-section; (**b**,**c**) are the distribution of creep cavities; (**d**) is the magnified graph of corresponding creep cavities in (**c**).

To further investigate the creep damage mechanism, the microstructures of BM and WM specimens after creep failure were observed by SEM. Specifically, the fractured BM and WM specimens were longitudinally sectioned, polished, and etched. Figure 9a displays the overall micrograph of the longitudinal cross-section of the BM sample, and Figure 9b–d shows the microstructures of the BM near the fracture tip. Obvious necking is observed in Figure 9a. By carefully observing the microstructures at different regions, it is found that a number of creep cavities with various size primarily initiate at carbide particles in the grain interior, as shown in Figure 9b–d. Furthermore, the growth of creep cavities is parallel to the loading direction, indicating that the applied stress promotes the coalescence of cavities.

**Figure 10:**
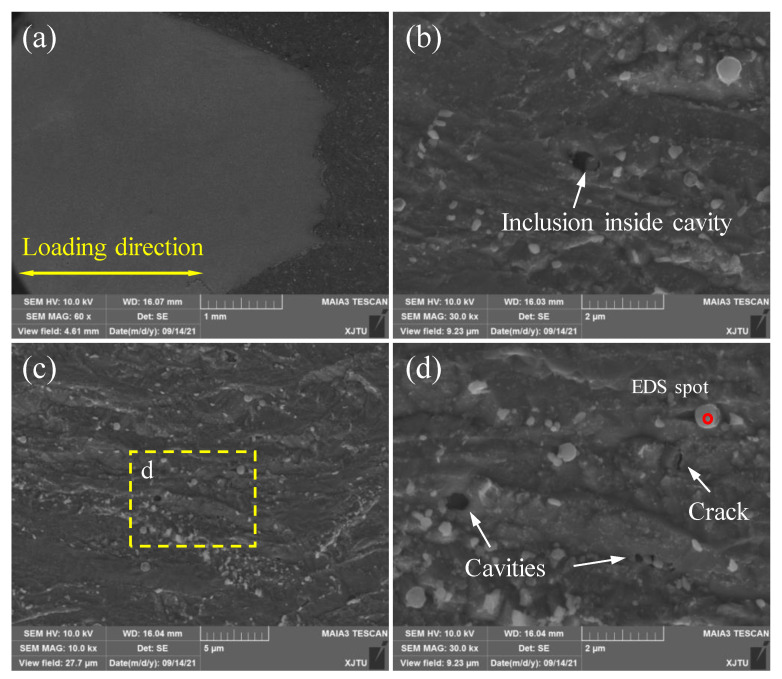
SEM images of the main damage zone of creep-ruptured WM specimen at 550 °C. (**a**) The overall micrograph of the longitudinal cross-section; (**b**,**c**) are the distribution of creep cavities; (**d**) is the magnified graph of corresponding creep cavities in (**c**).

**Figure 11:**
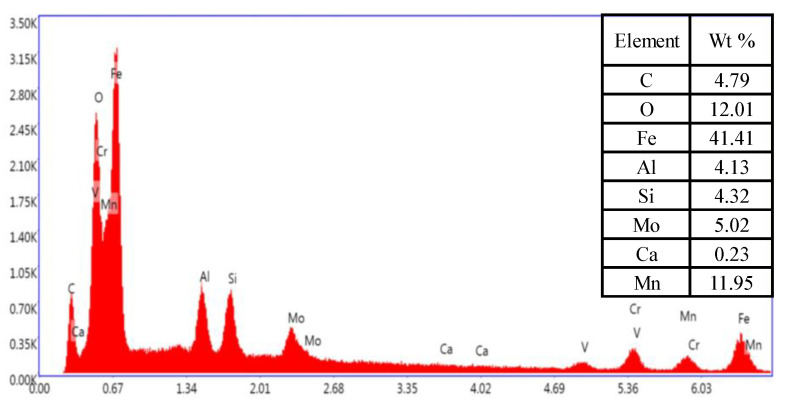
Energy-dispersive spectrum (EDS) profiles of the inclusion in WM marked in Figure 10d.

Figure 10 shows the microstructures of the creep-ruptured WM sample near the fracture tip. An evident necking phenomenon is found in the fracture surface. Numerous creep cavities with an elliptical shape can be found in the material (see Figure 10b,d). However, these cavities not only initiated at carbide particles but also at inclusions, as shown in Figure 10b. The existence of inclusions in the WM leads to localized stress concentration, hence promoting the initiation and growth of cavities. The elements of the inclusion marked in Figure 10d were measured by an energy-dispersive spectrometer (EDS), and the results are shown in Figure 11. It can be seen that there are significantly high contents of O, Al, Si, and Mn of inclusions in the WM sample. In addition, a small crack with a length of about 1 μm was found inside the material. The propagation of the microcrack, which is perpendicular to the loading direction, also induces the creep fracture.

It is important to clarify the observed microstructural damage in order to understand the failure mechanism of the 2.25Cr1Mo0.25V steel and weld. It is known that creep damage is essentially caused by the combined effects of creep cavity nucleation, growth, and coalescence. A number of studies have investigated the creep cavitation phenomenon in various metals and alloys, and the results show that the nucleation of cavities occurs in the early creep process (primary or secondary stage) at some susceptible locations in the microstructure [27,28]. The nucleation of cavities leads to a significant increase in localized stress that exceeds the applied stress. Further growth of these cavities under stress can directly result in the tertiary stage and eventual failure of materials [28]. As for the locations of creep cavities, it has generally been observed that the initiation and coalescence of cavities frequently generate at grain boundaries and the triple points of grain boundaries due to stress concentration [20,29,30]. It has been also reported that second phase particles above a critical size serve as the preferred sites for creep cavity nucleation and coalescence, since the particles can lead to stress concentration upon the application of the applied stress and, therefore, promote cavity nucleation [31,32,33]. For instance, Xu et al. [32] performed quantitative analysis on second phase particles associated with cavity formation in a 9% Cr tempered steel after about 11,000 h of creep exposure, and the results showed that the mean size of Al_2_O_3_, MnS, and BN particles was found to be less than 1 μm. In this study, creep cavity formation mainly occurs at the second phase particles with a small size for 2.25Cr1Mo0.25V steel BM and WM. Specifically, the cavities are initiated primarily at carbides in the BM, and the growth of cavities is parallel to the loading direction. In addition to carbides, the inclusion particles in the WM also act as the preferential sites for cavity nucleation. The growth and coalescence of the cavities initiated at second phase particles is further assisted by the applied stress and eventually results in creep failure. Therefore, controlling the number of inclusions in the weld by modifying the welding procedure and heat treatment process is helpful for regulating the creep resistance of weld metal in 2.25Cr1Mo0.25V steel welded joints.

In a word, it can be concluded that the initiation and growth of creep cavities at the second phase particles, such as carbides and inclusions, are the main creep damage mechanism of 2.25Cr1Mo0.25V steel BM and WM samples. Moreover, results from this study indicate that the mismatch in the high-temperature tensile strength, ductility, and creep deformation rate in 2.25Cr1Mo0.25V steel welded joints needs to be considered for the design and integrity assessment of hydrogenation reactors.

## 4. Conclusions

In the present study, the high-temperature tensile and creep deformation behaviors of the BM and WM in a 2.25Cr1Mo0.25V steel weldment used for the manufacture of a hydrogenation reactor were investigated experimentally, paying particular attention to its operating temperature range of 350–500 °C. Based on the obtained results, the following conclusions can be drawn:

(1) The uniaxial tensile tests in the temperature range of 350–550 °C reveal that the WM has higher strength and lower ductility than the BM. Moreover, both materials show a continuous softening behavior with an increase of testing temperature. Specifically, increasing temperature from 350 to 550 °C causes a 21.2% and 18.2% reduction in average UTS for BM and WM specimens, respectively.
(2) The short-term creep tests at 550 °C show that the creep curves of both the BM and WM have three distinct creep stages, including the primary creep stage, the secondary creep stage, and the tertiary creep stage. Moreover, the WM has a higher creep resistance than the BM. The creep damage mechanisms of both the BM and WM are the initiation and coalescence of creep cavities at the second phase particles.

## Figures and Tables

**Table 1:** Chemical composition of 2.25Cr1Mo0.25V steel base metal (BM) and weld metal (WM) (all elements are in wt.%).

Element	C	Si	Mn	P	S	Cr	Mo	V	Al	Ni	Cu
BM	0.15	0.10	0.54	0.009	0.01	2.30	0.98	0.30	0.05	-	-
WM	0.12	0.22	1.07	0.004	0.004	2.45	1.03	0.42	-	0.03	0.11

**Table 2:** Tensile properties of 2.25Cr1Mo0.25V steel BM and WM at high temperatures.

Material	Temperature (°C)	σ_Y_ (MPa)	σ_TS_ (MPa)	EL (%)	RA (%)
BM	350	454.0 ± 4.5	545.0 ± 5.3	17.7 ± 0.9	76.9 ± 1.5
BM	450	436.6 ± 5.7	507.3 ± 3.2	17.9 ± 2.0	76.2 ± 0.7
BM	550	391.5 ± 2.1	430.0 ± 0.4	19.7 ± 0.9	81.5 ± 1.5
WM	350	473.0 ± 14.4	550.3 ± 6.4	17.1 ± 1.1	70.7 ± 2.3
WM	450	448.2 ± 9.9	521.5 ± 7.8	18.1 ± 0.3	70.3 ± 2.0
WM	550	414.3 ± 4.7	450.3 ± 4.5	19.1 ± 0.5	79.6 ± 0.8

Note: σ_Y_, σ_TS_, EL, and RA are the yield strength, tensile strength, elongation, and reduction of area, respectively.

## Data Availability

The data used to support the findings of this study are available from the corresponding author upon request.
